# Medial Reduction in Sesamoid Position after Hallux Valgus Correction Surgery Showed Better Outcome in S.E.R.I. Osteotomy than DCMO

**DOI:** 10.3390/jcm12134402

**Published:** 2023-06-30

**Authors:** Yeok Gu Hwang, Kwang Hwan Park, Seung Hwan Han

**Affiliations:** 1 College of Medicine, Ewha Womans University, Seoul Hospital, Seoul 07804, Republic of Korea; oshwang@ewha.ac.kr; 2 College of Medicine, Yonsei University, Severance Hospital, Seoul 03722, Republic of Korea; khpark@yuhs.ac; 3 College of Medicine, Yonsei University, Gangnam Severance Hospital, Seoul 06273, Republic of Korea

**Keywords:** hallux valgus, minimally invasive surgery, sesamoid position

## Abstract

Background: The purpose of the present study was to compare the degree of sesamoid reduction after hallux valgus correction between distal chevron metatarsal osteotomy (DCMO) and S.E.R.I. (simple, effective, rapid, and inexpensive) osteotomy, and to analyze the effects on the recurrence of hallux valgus. Methods: We retrospectively analyzed the foot radiographs of 60 feet (30 DCMO and 30 SERI) treated for hallux valgus from August 2013 to July 2017. Radiographic assessments were performed preoperatively, at early follow-up (at a mean of 3.1 months) and at the most recent follow-up (at a mean of 16.7 months). The location of the medial sesamoid was classified into seven stages, in accordance with the method described by Hardy and Clapham; stage IV or less was defined as the normal position for the medial sesamoid, and stage V or greater was defined as lateral displacement of the sesamoid. The pre- and post-operative hallux valgus angle, 1–2 intermetatarsal angle, and sesamoid position were compared between the two groups. Results: The mean follow-up period was 18.4 (12–36) months in the DCMO group and 15.0 (12–36) months in the S.E.R.I. group (*p* = 0.108). The radiologic results showed that the hallux valgus angles were not significantly different between the two groups preoperatively and at the early follow-up: preoperatively, they were 28.8 ± 7.7 in the DCMO group and 32.6 ± 9.5 in the S.E.R.I. group (*p* = 0.101), and they were 10.4 ± 4.0 and 8.7 ± 5.0 (*p* = 0.148) at the early follow-up, respectively. However, at the most recent follow-up, the DCMO group (13.9 ± 5.6) showed significantly higher hallux valgus angles than the S.E.R.I. group (10.4 ± 6.4, *p* = 0.030), and there were no differences between the recurrence of hallux valgus in the DCMO group (13%)and that in the S.E.R.I. group (10%) (*p* = 0.553). There were no significant differences in the 1–2 intermetatarsal angles between the two groups at the early follow-up (6.1 ± 2.5 vs. 4.8 ± 3.1, *p* = 0.082) and at the most recent follow-up (7.3 ± 2.9 vs. 6.6 ± 3.5, *p* = 0.408). After hallux-valgus-correction surgery, the stage change of the tibia sesamoid position from the preoperative stage to the initial follow-up was significantly larger in the S.E.R.I. group (−4.4 ± 1.4) than in the DCMO group (−3.4 ± 1.1) (*p* = 0.003); the changes from the preoperative stage to the last follow-up were also significantly larger in the SERI group (−3.3 ± 1.7) than in the DCMO group (−2.4 ± 1.5) (*p* = 0.028); however, the changes from the initial follow-up to the last follow-up showed no significant differences between the two groups (+1.0 ± 1.1 in the DCMO group vs. +1.1 ± 1.2 in the S.E.R.I. group) (*p* = 0.822). The medial sesamoid was laterally subluxated in all the preoperative cases in the DCMO and S.E.R.I. groups. The lateral subluxation of the tibia sesamoid was more frequently observed in the DCMO group (four cases, 13%) than in the S.E.R.I. group (0 cases, 0%) (*p* = 0.038) at the early follow-up. Conclusion: In conclusion, our results demonstrated that the S.E.R.I. procedure is superior to DCMO in decreasing the hallux valgus angle and showed that the early post-operative reduction in the sesamoids can be a risk factor for the recurrence of hallux valgus.

## 1. Introduction

The main goals of the surgical correction of hallux valgus are the morphological and functional improvement of the first metatarsophalangeal joint and the first ray, the correction of the pain source over the medial exostosis, and the re-arrangement of the first metatarsophalangeal joint [1,2,3].

Out of more than 130 surgical procedures, distal chevron osteotomy for mild-to-moderate deformity is clearly approached the procedure of choice for many surgeons and has shown promising results [3,4,5]. Furthermore, S.E.R.I. (simple, effective, rapid, and inexpensive) osteotomy is a minimally invasive form of surgery, also known as Bosch osteotomy, and was shown by several authors to have the advantages of percutaneous techniques [6,7,8,9,10,11]. Minimally invasive osteotomy can result in substantial enhancement of the sesamoid position, which plays a critical role in preventing the recurrence of hallux valgus [12]. Several studies of S.E.R.I. have shown good results, with low rates of complications such as avascular necrosis of the metatarsal head or pseudoarthrosis, and low recurrence rates [13,14,15].

Sesamoid subluxation away from the head of the first metatarsal (MT) is usually indicative of hallux valgus deformity [16]. The position of the medial sesamoid (MS) in relation to the head of the first metatarsal and its correlation with HV deformity according to osteotomy methods has never been studied before. The lateral subluxation of the sesamoid bones may be strongly linked to the recurrence of hallux valgus deformity after corrective surgery [12]. The aim of our study was to compare the degree of sesamoid reduction in relation to the first metatarsal head after hallux valgus correction between distal chevron metatarsal osteotomy (DCMO) and S.E.R.I. osteotomy. We also evaluated the effects on the recurrence of hallux valgus deformity retrospectively.

## 2. Materials and Methods

Our institutional review board approved this study. Between August 2013 and July 2017, sixty consecutive patients (sixty feet) with an age of twenty years or more who had symptomatic hallux valgus deformity (a hallux valgus angle of ≥25° with subluxation of the first metatarsophalangeal joint or an intermetatarsal angle of ≥12°) were treated with two different surgical procedures, distal osteotomy of the first metatarsal bone in conjunction with distal soft tissue release (DSTR), at our institution. All patients could be followed up for at least one year. Patients with arthritis of the first metatarsophlangeal joint, as well as those with mild hallux valgus deformity and a history of corrective osteotomy, were excluded. Surgical treatment was performed in a ratio of 1:1. All procedures were performed by the senior author alone (S.H.H). Subjects were excluded from the study if they had an absence or hypoplasia of the medial sesamoid and if they had previously undergone hallux surgery.

Changes in the hallux valgus angle (HVA), intermetatarsal angle (IMA), and sesamoid position over time were analyzed by comparing values measured preoperatively, at early follow-up, and at the time of the most recent follow-up in both groups. In addition, cutoff values for recurrence were determined for each radiographic parameter, and the relative risks of recurrence, as indicated by preoperative and post-operative radiographic parameters, were determined.

Two foot-and-ankle surgeons who were independent of the operative team and were blinded to the outcome performed measurements of the radiographic parameters. Measurements were repeated 2 weeks later.

The HVA was defined as the angle between the longitudinal axis of the first metatarsal and that of the proximal phalanx. The IMA was defined as the angle between the longitudinal axis of the first and that of the second metatarsal. The longitudinal axis of the first metatarsal was defined as the line connecting the center of the proximal articular surface of the first metatarsal to the center of the first metatarsal head [17].

The longitudinal axis of the proximal phalanx and that of the second metatarsal was defined as the line connecting the centers of the proximal and distal ends of the diaphysis [17]. Sesamoid position was defined as the position of the medial sesamoid in relation to the longitudinal axis of the first metatarsal and was graded from 1 to 7 [18].

### 2.1. Operative Technique

#### 2.1.1. DCMO

A standard medial approach was taken to the first metatarsophalangeal joint, involving a longitudinal midline creating a 60-degree V-shaped capsulotomy followed by the removal of the medial eminence of the first metatarsal head [19]. We performed an intra-articular lateral capsular release by passing a number-11 knife blade horizontally between the sesamoids and the plantar aspect of the metatarsal head [20]. Osteotomy centered on the first metatarsal was performed in the same plane as the incision. The distal fragment was shifted by approximately 33% of its length, and its position was secured using a 1.6 mm Kirschner wire. With a distal metatarsal osteotomy, as well as medial capsulorrhaphy with absorbable sutures to correct the hallux, the osteotomy was then fixed with one headless compression screw. In cases in which correction of HVA was deemed necessary following metatarsal osteotomy, we opted to perform the Akin procedure concurrently. Furthermore, if symptoms were observed on the plantar aspect of the second MTP joint during preoperative evaluation, Weil osteotomy was also conducted.

#### 2.1.2. S.E.R.I. Osteotomy

Surgeries involved two surgeons, and they were performed under spinal or regional anaesthesia, with patients in supine position, using a tourniquet. The procedure began with application of a varus force for 2 to 3 min on the first MTP joint to stretch the lateral capsule, and surgery was performed using a medial approach through a one-centimeter medial incision just proximal to the medial eminence, at the level of the neck of the first metatarsal bone, exposing the neck of the metatarsal bone with two small retractors [14]. The osteotomy was performed with an oscillating saw, with the degree of blade inclination determined as part of the preoperative planning. The degree of inclination was based on the decision to lengthen or shorten the metatarsal bone. If the length of the metatarsal bone was to be maintained, the osteotomy was performed perpendicular to the axis of the foot. Otherwise, the osteotomy was performed at an inclination of up to 15°. Care was taken not to violate the lateral cortex with the saw blade, and the final cut was made with an osteotome to preserve the lateral periosteum. A 2 mm Kirschner wire is inserted to stabilize the osteotomy., which was advanced in retrograde fashion through the incision, close to the bone and along the length of the large toe until it protruded the skin at the distal end of the toe from the medial side, where it was withdrawn until its proximal end reached the osteotomy line. The K-wire was then advanced proximally to the osteotomy level inside the bone through the osteotomy site into the diaphyseal channel of the first metatarsal bone up to the base of the metatarsal For this procedure. Radiography was performed intraoperatively with a mini C-arm to confirm the displacement and repositioning of the sesamoids under the metatarsal head. If required, closing-wedge osteotomy was performed at 5 mm distal to the proximal phalanx base. The medial capsule was repaired using absorbable sutures without tension.

### 2.2. Post-Operative Care

Hard-sole shoe was applied for four weeks post-operatively. Weight bearing on the heel was allowed on the day after surgery. Kirschner wires used for SERI osteotomy were removed during the fifth post-operative week. Headless compression screw for DCMO was not removed. Full weight bearing on the first ray was not allowed until the sixth post-operative week. Progressive rehabilitation with passive and active exercises were recommended.

### 2.3. Radiographic Assessments

All radiographs were performed at a single facility and according to the same radiographic protocol. Weight-bearing dorsoplantar radiographs were obtained at multiple time points, including preoperatively, at 6 weeks, 3 months, 6 months, 12 months, and at the final follow-up. For this study, we utilized the preoperative, 3-month (early follow-up), and last follow-up radiographs. The radiographs were retrieved by a picture-archiving and communication system (PACS) (IMPAX; Agfa HealthCare). Radiographic measure-ments were conducted using PACS software by two foot-and-ankle surgeons, who were independent of this study.

### 2.4. Clinical Assessment

To evaluate subjective patient satisfaction with the procedure, we asked the patients for their subjective opinions, which we categorized, using the Coughlin scale [21], as “excellent,” “good,” “fair,” or “poor.” We also evaluated the post-operative complications in terms of recurrence, hallux varus, and avascular necrosis. The recurrence of hallux valgus was defined as a HVA of more than 20 degrees [22,23].

### 2.5. Data Collection and Statistical Analysis

Clinical and operative data collected for the study included patients’ ages, sexes, weights, heights, and whether the deformity was of the left foot or right foot. Categorical variables are expressed as the number and percentage of patients, and continuous variables are expressed as mean ± standard deviation (SD). Differences between pre- and post-operative categorical variables were analyzed by chi-square test, and differences between pre- and post-operative continuous variables were analyzed by paired *t* test. To compare the means of two independent groups (DCMO versus S.E.R.I. groups), an independent *t*-test was used. Statistical analysis was performed using the IBM SPSS version 24 software for Windows (IBM Corp., Armonk, NY, USA). A *p* value of <0.05 was considered statistically significant.

## 3. Results

### 3.1. Clinical Results

The comparative demographics of the two groups are shown in Table 1. No intergroup difference was evident. All of the patients had significantly improved at their most recent follow-up to excellent and good (32 and 28 feet, retrospectively), while preoperatively, the improvements were poor and fair (51 and 9 feet, retrospectively) in terms of subjective patient satisfaction.

### 3.2. Radiographic Results

The hallux valgus and intermetatarsal angles in the DCMO group and S.E.R.I. group are shown in Table 2. The average pre-operative intermetatarsal angle in the S.E.R.I. group was greater than the average intermetatarsal angle in the DCMO group (*p* = 0.04). In the DCMO group, the average hallux valgus angles at the time of the most recent follow-up were significantly greater than those in the S.E.R.I group (*p* = 0.03). There was a significant improvement in the HVA on most of the recent post-operative radiographs. Several articles have defined recurrence after hallux valgus correction as a HVA of ≥20°; thus, recurrence in the current study was also defined as a HVA of ≥20° [22,23]. Recurrence was observed in four feet (13%) in the DCMO group and three feet (10%) in the S.E.R.I. group at the time of the most recent follow-up. No significant differences with respect to the IMA at all time points after surgery were observed between the groups. Furthermore, avascular necrosis was not observed in either group.

### 3.3. Medial Sesamoid Position

The grade and the grade difference of the distribution of the medial sesamoid bone position are shown in Table 3. We chose Hardy and Clapham’s method because it provides a more detailed and precise classification than the traditional Mann-and-Coughlin parameters. On the basis of the results regarding the sesamoid positions in this study, we defined grade IV or less as the normal sesamoid position and grade V or greater as lateral displacement of the sesamoid [12]. At the time of the early follow-up, twenty-six feet were in the DCMO group and thirty in the S.E.R.I. group. At the time of the most recent follow-up, seventeen feet were in the DCMO group and twenty-four in the S.E.R.I. group. We used the median-input data as a method for handling missing values.

In terms of the grade difference in the position of the medial sesamoid bone after surgery, that is, the degree of the reduction in the sesamoid bone at the time of the early and most recent post-operative follow-up, the S.E.R.I. group had a significantly greater degree of sesamoid reduction than the DCMO group (*p* = 0.003, *p* = 0.028, retrospectively). However, between the early and most recent follow-up, there was no significant difference between the two groups in the grade difference of the medial sesamoid bone.

All the cases of the lateral displacement of the medial sesamoid bone were in the DCMO and S.E.R.I. groups before surgery, but at the early follow-up, the DCMO group had significantly more lateral displacement, and at the most recent follow-up, the DCMO group was almost significantly different from the S.E.R.I. group.

## 4. Discussion

To our knowledge, this study is the first to report a comparison of sesamoid reduction between DCMO and S.E.R.I. operations. In several articles, the location of the sesamoid medially with rotation in hallux valgus was a predictor of recurrence [12,22,24,25,26,27]. The lateral displacement of the sesamoids is believed to be related to the post-operative recurrence of hallux valgus; however, we are not aware of any previous studies in which the relationship between two different surgeries and the recurrence of hallux valgus on plain radiograph in the early and most recent follow-up was investigated. We suggest that the results from the analysis of early post-operative radiographs can be used to predict recurrence with the laterally displaced sesamoid in the radiographic parameters. In addition, the medial sesamoid bones in the feet in the DCMO group at the time of the early follow-up had a greater risk of recurrence of hallux valgus than those in the S.E.R.I. group.

The present study identified a relationship between the reduction loss of the sesamoids and radiographic evidence of the recurrence of hallux valgus. The prevalence of the recurrence of hallux valgus, as reported by several articles, ranges from 4% to 25% [28,29,30]. Several articles have defined recurrence after hallux valgus correction as a HVA of ≥20°; thus, recurrence in the current study was also defined as a HVA of ≥20° [22,23,31]. This study demonstrates that there were no differences in terms of recurrence between the two different surgical procedures in the most recent follow-up. The results showed that percutaneous distal metatarsal osteotomy (a.k.a S.E.R.I. operation) is sufficient to achieve good correction of hallux valgus deformities, in accordance with previously reported results in the literature [14,32].

Hardy and Clapham [18] established a seven-grade classification system using the medial sesamoid and the mid-axis of the first metatarsal as the reference line to evaluate sesamoid displacement. They found that the grade of sesamoid displacement ranged from I to VI, with 90% showing a grade of III or less in normal feet, whereas the grades ranged from I to VII, with 88% showing a grade of IV or greater in feet with hallux valgus preoperatively. In the present study, none of the cases were classified as grade IV or less. All sixty feet in the two different groups were classified as grade V or greater. The distributions of the sesamoid positions with hallux valgus in the present study were somewhat matched to those in the study by Hardy and Clapham. We support the notion that the cutoff point between normal and abnormal sesamoid positions on plain radiographs is grade IV on the basis of our radiographic results.

**Figure 1:**
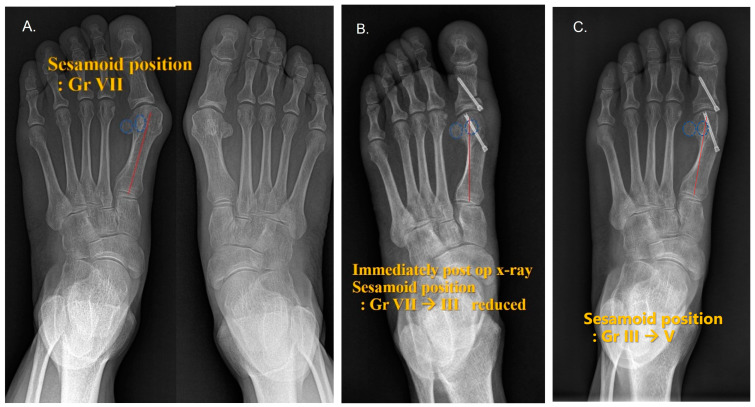
Radiological appearance and anteroposterior radiograph of a 57-year-old female after distal chevron metatarsal osteotomy (DCMO) for moderate hallux valgus. (**A**) Pre-operative, sesamoid position VII; (**B**) immediately post –operative, sesamoid position III; and (**C**) one year after the surgery, sesamoid position V. The correction was not maintained, but the patient was pain- free while bearing weight.

**Figure 2:**
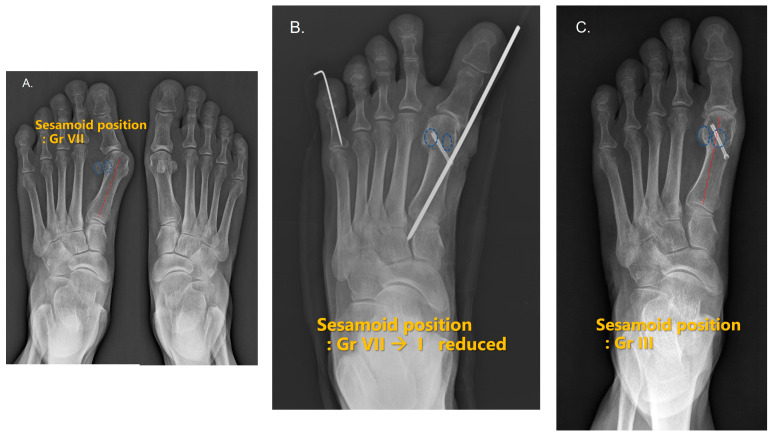
Radiological appearance and anteroposterior radiograph of a 66-year-old female after S.E.R.I. (simple, effective, rapid, and inexpensive) osteotomy for moderate hallux valgus. (**A**) Pre-operative, sesamoid position VII; (**B**) immediately post –operative, sesamoid position I; (**C**): one-and-a-half years after the surgery, sesamoid position III. The correction was not maintained, but HVA was 35° to 10° and IMA was 16° to 4° (preoperative and recent follow-up, respectively).

Conventionally, on a weight-bearing AP radiograph, the sesamoid can appear subluxated, depending on whether the erosion of the intersesamoidal ridge is present. R. Ramdass et al. [27] suggested that the relocation of the relatively mobile first metatarsal over the relatively immobile sesamoids is the cause of this phenomenon. In this study, these measurements did not take a tangential sesamoid view in determining the sesamoid position. Thus, the true sesamoid position in the AP planes may not have been accurate. The concept of sesamoid positions may be considered as a different anatomical reference from weight-bearing plain radiographs (Figure 1 and Figure 2).

The present study is limited by its retrospective nature and the short-term follow-up data used. Our follow-up period was relatively short (18.4 months and 15.0 months, retrospectively). However, percutaneous metatarsal osteotomy was reported to be a safe and effective technique. Jeyaseelan and Malagelada reported that for minimally invasive hallux valgus surgery, the radiological and clinical outcomes seem to be comparable to those of open procedures [33]. A long-term follow-up of S.E.R.I should be conducted. Furthermore, we did not exclude cases that included the Akin procedure. This additional procedure may have affected the radiological results. In addition, in the patients who underwent S.E.R.I., the procedures were performed without an additional procedure; therefore, it seems unlikely that the additional procedures affected the radiographic results in the present study. Furthermore, we did not consider the factor of first-ray hypermobility in relation to hallux-valgus-deformity recurrence. We believe that there is no direct impact of hypermobility on the recurrence of hallux valgus. This study is also limited in that the position of the sesamoid bone on weight-bearing AP radiography may not accurately represent its true anatomical position due to various factors, mentioned above. Therefore, this study proposes the need for new anatomical criteria regarding the observed position of the sesamoid bone on weight-bearing AP radiography.

Seng et al. [24] reported that the reduction in and restoration of the sesamoid position improved significantly in line with the surgeon’s experience for this procedure. Their study was conducted over a long time period (from August 2013 to July 2017). Generally, S.E.R.I. surgery began to be used more recently than DCMO surgery, and this factor may have influenced the outcome. In the future, it is necessary to conduct a study on whether the learning curve according to the date of surgery is a risk factor for the recurrence of hallux valgus. However, we believe that patients corrected with minimally invasive surgery of the first metatarsal bone might benefit from this simple, effective, rapid, and inexpensive procedure for the treatment of hallux valgus.

In conclusion, our results demonstrated that the S.E.R.I. procedure is superior to DCMO in terms of the decrease in hallux valgus angle and showed that the early post-operative reduction loss of the sesamoids can be a risk factor for the recurrence of hallux valgus. Future research with radiographic and comparative studies to reveal the role of percutaneous metatarsal osteotomy in the treatment of hallux valgus is needed.

## Figures and Tables

**Table 1:** Demographics of two groups.

	DCMO (n = 30, 50%)	S.E.R.I. (n = 30, 50%)	*p* Value *
Age (years)	50.4 ± 15.4	56.0 ± 12.4	0.140
Gender (female: male)	26:4	27:3	0.999
Right:left	18:12	16:14	0.602
Body-mass index, kg/m^2^	24.0 ± 3.0	22.8 ± 2.8	0.017
Follow-up period (Months)	18.4 ± 6.0	15.0 ± 9.4	0.108

* Independent *t* test. Mean ± standard deviation.

**Table 2:** Comparisons of radiological outcomes between DCMO group and S.E.R.I. group.

	DCMO (n = 30, 50%)	S.E.R.I. (n = 30, 50%)	*p* Value
Hallux valgus angle (°)			
Pre-op	28.8 ± 7.7	32.6 ± 9.5	0.101
Early follow-up	10.4 ± 4.0	8.7 ± 5.0	0.148
Most recent follow-up	13.9 ± 5.6	10.4 ± 6.4	**0.030**
Most recent follow-up ≥20°	4(13%)	3(10%)	0.553
Intermetatarsal angle (°)			
Pre-op	11.9 ± 2.6	13.5 ± 3.1	**0.040**
Early follow-up	6.1 ± 2.5	4.8 ± 3.1	0.082
Most recent follow-up	7.3 ± 2.9	6.6 ± 3.5	0.408

Values are expressed as no. (%) unless otherwise indicated. SD, standard deviation. Boldface indicates statistical significance (*p* < 0.05).

**Table 3:** Comparisons of distribution of the medial sesamoid bone positions between DCMO group and S.E.R.I. group.

	**DCMO (n = 30, 50%)**			**S.E.R.I. (n = 30, 50%)**		
**Grade**	**Pre-Operative**	**Early Follow-Up**	**Most Recent Follow-Up**	**Pre-Operative**	**Early Follow-Up**	**Most Recent Follow-Up**
I		3 (10%)	1 (3%)		16 (53%)	7 (23%)
II		10 (33%)	5 (17%)		4 (13%)	3 (10%)
III		9 (30%)	6 (20%)		5 (17%)	8 (27%)
IV		4 (13%)	5 (17%)		5 (17%)	6 (20%)
V	10 (33%)	4 (13%)	10 (33%)	6 (20%)		5 (17%)
VI	2 (7%)		3 (10%)	7 (23%)		1 (3%)
VII	18 (60%)			17 (57%)		
				**DCMO** **(n = 30, 50%)**	**S.E.R.I.** **(n = 30, 50%)**	***p* value**
**Grade difference**						
Pre-op to early follow-up				−3.4 ± 1.1	−4.4 ± 1.4	0.003 *
Pre-op to most recent follow-up				−2.4 ± 1.5	−3.3 ± 1.7	0.028 *
Early to Most recent follow up				1.0 ± 1.0	1.1 ± 1.2	0.822 *
Laterally displaced sesamoid						
Pre-op				30 (100%)	30 (100%)	0.999 †
Early follow-up				4 (13%)	0 (0%)	**0.038** †
Most recent follow-up				13 (42%)	6 (20%)	0.052 †

* Independent *t*-test for the comparison between groups. † Chi-square test for the comparison between groups.

## Data Availability

Data is unavailable due to ethical restrictions, however, if there are any specific requests, we can provide them individually if needed.
